# Influence of Professional Identity on the E-Learning Adaptability Among Chinese Nursing Students During COVID-19

**DOI:** 10.3389/fpubh.2021.754895

**Published:** 2022-01-27

**Authors:** Huixiao Wang, Mingying Yang

**Affiliations:** ^1^Department of Rehabilitation Medicine, The Second Affiliated Hospital of Kunming Medical University, Kunming, China; ^2^Department of Nursing, The Second Affiliated Hospital of Kunming Medical University, Kunming, China

**Keywords:** computer-assisted instruction, professional identity, adaptability, students, COVID-19, E-learning, nursing

## Abstract

**Background:**

The traditional face-to-face education methods have been altered to E-learning due to the outbreak, and the E-learning adaptability of nursing students will directly affect the effectiveness of online education. The professional identity of nursing students refers to the positive perception, evaluation, and emotional experience of the nursing profession and identity to be undertaken, which may affect the E-learning adaptability of nursing students during the coronavirus disease 2019 (COVID-19). This study aimed to explore the impact of professional identity on the E-learning adaptability of the Chinese nursing students during COVID-19.

**Methods:**

This study was conducted in three medical schools in Yunnan Province, China from August to October 2020. Data collection consisted of three sections: participants' characteristics, learning adaptability, and professional identity.

**Results:**

A total of 585 nursing students had a moderate level of E-learning adaptability. There was a positive correlation between E-learning adaptability and professional identity (*r* = 0.316~0.505, *p* < 0.001). In addition, the professional identity was associated with predictors of the E-learning adaptability among nursing students (*p* < 0.001).

**Conclusion:**

There was a moderate level of E-learning adaptability among the Chinese nursing students during the COVID-19 crisis. Enhancing the professional identity is critical in improving the E-learning adaptability among nursing students.

## Introduction

Coronavirus disease 2019 (COVID-19) has spread widely throughout China and the world, which brings lots of challenges to the educational work in institutions of higher learning (1, 2). The traditional educational methods are altered to E-learning because the conventional face-to-face education is considered as an opportunity for the virus to spread. E-learning has been adopted in most colleges and universities to ensure learning from the beginning of the outbreak. In 2020, the Ministry of Education issued a new regulation that colleges and universities should take full advantage of E-learning platforms to ensure teaching progress and quality (3). Since then, E-learning has been widely applied as a vital part of the learning pattern.

E-learning, also known as online education, refers to the use of the Internet, computers, smartphones, and other electronic resources to acquire and disseminate knowledge, such as network education, digital learning, interactive learning, and computer-assisted teaching (4, 5). Compared with the traditional classroom teaching pattern, the network teaching platform breaks the limitation of time and space. However, students need the spirit of independence, initiative, and consciousness in the progress of E-learning. E-learning adaptability directly affects the effectiveness of online education (6). E-learning adaptability is the capacity to adjust behaviors, thoughts, and feelings in response to the variable, uncertain, and unexpected situations and circumstances in the process of online learning. It realizes the changed process of balance between the individual and the learning environment (7). Adaptability is an essential academic and personal development capacity among students (8). The better the E-learning adaptability of students, the better the learning effect (9). Conversely, learning maladjustment may adversely affect their learning efficiency and the occupation prospects for development (10, 11). Nowadays, many universities have recognized the importance of E-learning as a core element of their learning system. E-learning adaptability of students belongs to the middle level of the whole. More than half of students showed low learning autonomy and satisfaction in web-based learning in China (12, 13).

The professional identity of nursing students refers to the positive perception, evaluation, and emotional experience of the nursing profession and identity to be undertaken. It determined the career choice of nursing students and the stability of the nursing team to some extent. The formation of professional identity was a dynamic process for a nurse. It started at the professional education stage and then developed, fluctuated, and accompanied the entire career. The role of nursing students was a crucial period for the formation of professional identity. Nursing students with a stronger professional identity were more likely to remain in their work posts and improve the quality of care in the long run (14–16). However, there is no relevant research on whether the professional identity of nursing students will directly affect their learning adaptability.

So, in this study, we aim to (1) assess the situation of E-learning adaptability and professional identity of Chinese nursing students during the COVID-19; (2) identify the association between E-learning adaptability and professional identity; and (3) determine the influential factors of the E-learning adaptability among the Chinese nursing students.

## Materials and Methods

### Study Design

This study was a cross-sectional study on nursing students enrolled in three medical schools in Yunnan province using the STROBE guideline for cross-sectional studies. All theoretical courses through online learning of this semester during the epidemic prevention and control from March to July 2020. E-learning methods include the live, record, or sometimes both. It involves the online platform of Rain classroom, Tencent Meeting, and Zoom app. It is essential for implementing online education with computers, virtual reality devices, mobile phones, and personal digital assistants. Institution of higher education has conducted training on the use of the online teaching platform and online collective preparation of instruction to teaching staff. Teachers adjust the curriculum plan in time according to the characteristics of online teaching. The teaching and research section conducts a rational examination and evaluation system for E-learning courses.

### Participants

Participants were nursing students enrolled in three medical schools in Yunnan Province. Students who met the following criteria were included in this study: (1) currently studying in the school of nursing; (2) participated in E-learning from March to July 2020; (3) participants had no cognitive impairment or obvious language barriers and provided their informed consent to participate in this study. International students were excluded. Inclusion and exclusion criteria were stated in the content of the invitation to participate link.

### Measurement

The questionnaire consisted of three sections: sociodemographic characteristics, learning adaptability, and professional identity.

The sociodemographic characteristics form was designed based on previous studies and the opinion of two nursing specialists, such as age, gender, educational level, residence, household incomes per capita (CNY), duration of classes in a week, preferred teaching method, and network environment.

E-learning adaptability was measured for the undergraduates' learning adaptability questionnaire (ULAQ),and compiled and validated in China by Xu (17). The questionnaire consists of 55 items and two dimensions of learning motivation and learning behavior. Each item was rated on a 5-point Likert scale ranging from 1 (not at all) to 5 (exactly right), such as “1” is poor, “2” is middle and lower, “3” is middle, “4” is middle and upper, and “5” is excellent. Total and two dimensions scores were calculated by taking the average across all items within each dimension. The higher the score, the better the learning adaptability of nursing students. This questionnaire has good reliability and validity. The internal consistency coefficient of total scores and two dimensions were 0.891, 0.789, and 0.864. The correlation coefficient between total scores and two dimensions was 0.783 and 0.893. ULAQ was used to assess the learning adaptability among college students in previous studies (18, 19).

Professional identity was measured using the Professional Identity Questionnaire for Nurse Students (PIQNS), developed and validated by Chinese scholars (20). The questionnaire consists of 17 items, with five dimensions respectively: professional self-image (six items), the benefit of retention and risk of turnover (four items), social comparison and self-reflection (three items),independence of career choice (two items), and social modeling (two items). Each item was rated on a 5-point Likert scale ranging from 1 (not at all) to 5 (exactly right). Total and each dimension scores were calculated by taking the average across all items within each dimension; a higher score indicated a higher level of professional identity. The Cronbach's alpha and the split-half reliability of PIQNS were 0.827 and 0.842, respectively.

### Statistical Collection

As a result of the pandemic outbreak, we conducted a web-based online survey to reduce face-to-face interactions. Therefore, data were collected through a professional online questionnaire platform. After they signed written informed consent, a text message containing the URL of the online survey was sent to all the students. At the start of the online study, we validated our participants by asking them about their major and college. Participant information was electronically encoded for data storage, and the computer of researcher was password-protected to prevent unauthorized access. The system would remind respondents of missing responses before submission, and only full completed questionnaires were allowed to submit. A total of 600 questionnaires were received in this online survey, and 15 invalid questionnaires with incorrect information were excluded. The remaining 585 valid questionnaires were valid, with an effective rate of 97.5%.

### Statistical Analysis

All statistical analyses were performed using SPSS Statistics 25.0 (IBM Corp, Armonk, NY, USA). Data were expressed as means ± SD. We used Kolmogorov–Smirnov to assess the normality of the distribution of continuous variables. Descriptive statistics were used for the participant characteristics of E-learning adaptability and professional identity among nursing students. Independent samples *t*-tests and one-way ANOVA tests were conducted to further analyze the ULAQ scores by sociodemographic variable. Correlation analysis was used to determine the relationship between total dimensions and each dimension of professional identity and E-learning adaptability. The magnitudes of correlations are classified as follows: ≤ 0.25 very low; 0.26 ≤ *r* ≤ 0.49 low; 0.50 ≤ *r* ≤ 0.69 moderate; 0.70 ≤ *r* ≤ 0.89 high; and 0.90 ≤ *r* <1 very high. Multiple linear regression was used to analyze the influential factors of E-learning adaptability. All tests were two-tailed, with a significance level of *p* < 0.05.

## Results

### Characteristics of Participants

A total of 585 nursing students were included in this study, and the average score for age was 20.17 ± 1.38. Participant characteristics are presented in Table 1. Most respondents in this study were women (89.91%) and undergraduate students (52.48%). Approximately 77.09% of respondents live in rural areas, and 63.93% reported a poor network environment. Household incomes per capita (CNY) of respondents mainly were around 1,000–3,000 yuan (57.78%). The duration of classes in a week of most respondents was 15–25 h (38.63%), followed by more than 25 h (33.85%), and <15 h (27.52%). About 70.77% of respondents preferred live as their teaching method in E-learning.

**Table 1:** Sociodemographic characteristics of the study participants (*N* = 585).

**Variable**		***N***	**%**
Gender	Male	59	10.09
	Female	526	89.91
Educational level	Undergraduate students	307	52.48
	Junior college students	278	47.52
Residence	Urban	134	22.91
	Rural	451	77.09
Household incomes per capita (CNY)	<1000	145	24.79
	1000–3000	338	57.78
	>3000	102	17.43
Duration of classes in a week	<15 h	161	27.52
	15–25 h	226	38.63
	>25 h	198	33.85
Preferred teaching method	Record	131	22.39
	Live	414	70.77
	Combination	40	6.84
Network environment	Stable	211	36.07
	Poor	374	63.93

### Descriptions of E-Learning Adaptability Among Nursing Students

Data shown in Table 2 clearly illustrated the E-learning adaptability among nursing students. Gender (*p* = 0.146) and household incomes per capita (CNY) (*p* = 0.159) were no statistical significance with E-learning adaptability. ULAQ score was significantly associated with the educational level of respondents (*p* < 0.001), residence (*p* < 0.001), duration of classes in a week (*p* < 0.001), preferred teaching method (*p* < 0.001), and network environment (*p* < 0.001). The highest ULAQ scores were from undergraduate (3.65 ± 0.47), living in urban areas (3.81 ± 0.67), 15–25 h courses per week (3.68 ± 0.41), combination teaching methods (3.76 ± 0.60), and stable network environment (3.58 ± 0.55) among the Chinese nursing students. *Post-hoc* analyses (Appendix Table 1) result from the comparison showed that 15–25 h courses per week scored better than <15 h (*p* < 0.001) and more than 25 h (*p* < 0.001), and <15 h scored better than more than 25 h (*p* = 0.007). A combination of both scored better than record (*p* = 0.001) and live (*p* = 0.002). Compared with the record (3.34 ± 0.57), live (3.40 ± 0.46) had higher ULAQ scores, but the difference was not significant (*p* = 0.499).

**Table 2:** Undergraduates' Learning Adaptability Questionnaire (ULAQ) scores among nursing students with different sociodemographic characteristic (*N* = 585).

**Variable**		**Mean (SD)**	**Statistical** **test**	***P***
Gender	Male	3.50 (0.51)	*t* = 1.457	0.146
	Female	3.40 (0.51)		
Educational level	Undergraduate students	3.65 (0.47)	*t* = 13.368	0.000***
	Junior college students	3.15 (0.42)		
Residence	Urban	3.81 (0.67)	*t* = 8.535	0.000***
	Rural	3.30 (0.38)		
Household incomes per capita (CNY)	<1000	3.35 (0.49)	*F* = 1.843	0.159
	1000–3000	3.44 (0.48)		
	>3000	3.41 (0.61)		
Duration of classes in a week^a^	<15 h	3.35 (0.39)	*F* = 55.291	0.000***
	15–25 h	3.68 (0.41)		
	>25 h	3.18 (0.61)		
Preferred teaching method^b^	Record	3.34 (0.57)	*F* = 10.892	0.000***
	Live	3.40 (0.46)		
	Combination	3.76 (0.60)		
Network environment	Stable	3.58 (0.55)	*t* = 5.93	0.000***
	Poor	3.32 (0.46)		

***Significant at 0.001.

*^a^15–25 h>(<15 h) (p < 0.001) and (> 25 h) (p < 0.001), (<15 h)>(>25 h) (p = 0.007).*

*^b^Combination >Record (p = 0.001) and Live (p = 0.002), Live > Record (p = 0.499).*

*ULAQ, Undergraduates' Learning Adaptability Questionnaire*.

### Correlation Analysis Between E-Learning Adaptability and Professional Identity of Nursing Students

Appendix Table 2 represents the dimensions and total scores of E-learning adaptability and professional identity for nursing students. The average score of E-learning adaptability was 3.41 ± 0.51, including two dimensions of learning motivation (3.34 ± 0.56) and learning behavior (3.42 ± 0.52). The average score of professional identity was 61.60 ± 10.06, it had five dimensions according to the ranking of total scores as follow: (1) professional self-image (22.5 ± 4.03), (2) benefits of retention and risk of turnover (13.54 ± 2.88), (3) social comparison and self-reflection (11.28 ± 2.15), (4) independence of career choice (6.39 ± 1.51), and social modeling (7.85 ± 1.60). There was a positive correlation between total dimensions and each dimension of professional identity and E-learning adaptability (*r* = 0.316~ 0.505, *p* < 0.001), which is shown in Table 3.

**Table 3:** The correlation between the E-learning adaptability and professional identity dimensions (*r*).

**Variable**	**Total** **scores**	**Professional** **self-image**	**Benefits of** **retention and** **risk of turnover**	**Social comparison** **and self-reflection**	**Independence of** **career choice**	**Social** **modeling**
Total scores	0.505***	0.453***	0.454***	0.384***	0.425***	0.357***
Learning motivation	0.504***	0.479***	0.468***	0.418***	0.413***	0.402***
Learning behavior	0.445***	0.402***	0.402***	0.342***	0.374***	0.316***

*r: Correlation coefficient.*

***Significant at 0.001.

### Factors Influencing the E-Learning Adaptability of Nursing Students

Multiple linear regression analysis is shown in Table 4. The E-learning adaptability was a dependent variable. The independent variables included the educational level, residence, duration of classes, preferred teaching method, network environment, and professional identity. These were significant predictive factors on the E-learning adaptability of nursing students (*F* = 85.327, *p* < 0.001).

**Table 4:** Multiple linear regression analysis for E-learning adaptability of nursing students (*N* = 585).

**Model**	**Unstandardized coefficients**		**Standardized** **coefficients**	***t***	***P***	**95% Confidence** **interval**
	**B**	**Std. Error**				
Constant	2.436	0.132		18.510	0.000***	2.178, 2.695
Education level (Ref. = Undergrauate student)						
Junior college students	0.269	0.031	0.264	8.636	0.000***	0.208, 0.331
Residence (Ref. = Urban)						
Rural	−0.253	0.037	−0.209	−6.908	0.000***	−0.325, −0.181
Duration of classes in a week (Ref. = 15–25 h)						
<15 h	−0.054	0.038	−0.047	−1.421	0.156	−0.127, 0.020
>25 h	0.163	0.036	0.151	4.541	0.000***	0.092, 0.233
Preferred teaching method (Ref. = Combination)						
Record	−0.183	0.064	−0.150	−2.851	0.005**	−0.309, −0.057
Live	−0.117	0.059	−0.104	−1.982	0.048*	−0.232, −0.001
Network environment (Ref. = Stable)						
Poor	−0.088	0.032	−0.083	−2.768	0.006**	−0.150, −0.025
Professional identity	0.019	0.002	0.375	11.702	0.000***	0.016, 0.022

*R = 0.736, R^2^ = 0.542, Adjusted R^2^ = 0.536, F = 85.327, p < 0.001.*

*Significant at 0.05, **Significant at 0.01, ***Significant at 0.001.

*Ref, Reference*.

## Discussion

### E-Learning Adaptability of Nursing Among the Chinese Nursing Students During COVID-19

With the transformation of teaching methods during the epidemic, the adaptability of online learning of college students has received extensive attention and research. In this study, the E-learning adaptability of nursing students was at a medium level, based on the total average ULAQ score (3.41 ± 0.51). Similar results were observed with Saudi Arabia (21) and Egypt (22) studies, which indicated that about half of the students showed a positive attitude toward E-learning and had some barriers. The effectiveness of e-learning programs in developing countries scores lower than in developed countries among medical students (23). Developed countries may have built and adapted the technological infrastructure for the transition of face-to-face to digital education. In contrast, developing countries are not fully prepared for the shift. The integration of information and communication technology in teaching and learning is still early in the education systems (24). Arthur-Nyarko (25), DePaul (26), and other researchers also reported that unstable network connections and the lack of computers in homes in rural areas are important factors affecting the progress and quality of online learning. The results are in keeping with our findings that nursing students who live in rural areas and have unreliable network connections have lower ULQA scores. At the same time, undergraduate, 15–25 h courses per week and combination of live and record among nursing students have better E-learning adaptability. There were no significant differences in gender and household incomes per capita. Almaiah (27), Singh (28) reported similar results. However, some suggest that affluent family is an essential factor of E-learning feasibility for nursing students, and women showed a more positive response toward E-learning than men in previous studies (29). Therefore, it is necessary to evaluate further and analyze the influential factors of the E-learning adaptability among nursing students. Based on the analysis results, strategies to improve the E-learning adaptability of students will be formulated.

### E-Learning Adaptability and Professional Identity

There was a positive correlation between E-learning adaptability and professional identity, and the higher the professional identity, the higher the E-learning adaptability. In the regression model, professional identity is an essential predictor of E-learning adaptability among the Chinese nursing students during COVID-19. Existing research showed that professional identity and learning burnout were negatively correlated (30). More than 60% of Chinese college students are often unclear about their future career goals and lack active academic participation (31, 32). Students often face difficulties due to the lack of a good learning attitude, such as the lack of self-control and indiscipline when self-isolated at home (33). Therefore, by helping the formation of professional identity in nursing students, learning adaptability will possibly be more developed in them. Professional identity is considered to construct and deconstruct through nursing education continuously (34, 35). It is suggested that colleges and universities pay attention to career guidance of nursing students, improve the online career planning curriculum, and guide nursing students to set up the correct values of career choice in the teaching management process. Additionally, teachers should help students to set up the learning goals of professional courses and complete online learning courses in a planned way. On the other hand, it strengthens the cultivation of professional identity among nursing students. Through guiding students to understand the value of nursing work in the learning process, stimulating nursing students demands for academic knowledge, and thus significantly promote the E-learning adaptability of nursing students.

### Factors Affecting the E-Learning Adaptability

Previous studies have shown similar findings that the higher the educational level, the higher the E-learning adaptability of nursing students. Lu (36) shows that the higher the educational level, the better the online learning self-efficacy and deep learning level. Undergraduate and junior college nursing students have different self-learning abilities, and different coping styles in solving significant learning problems might be one of the reasons causing the difference in E-learning adaptability. Similarly, Zhao (37) reported that postgraduates have better overall online learning adaptability than undergraduates due to stronger independent innovation thinking and more precise knowledge needs. Another study found that the learning burnout of junior college students is higher than undergraduate students (35). Therefore, it is necessary to fully evaluate the current situation of the problems and needs encountered by different educational background groups in the progress of online learning and to apply online tests intellectually to teach students according to their aptitude.

We observed that the residence and network environment were essential predictors of E-learning adaptability. Compared with living in the city, limited internet access, such as an unstable network environment and a lack of computers in homes in rural areas, affected the E-learning progress. Students from cities have better availability of Wi-Fi routers and dedicated rooms at home and better proficiency in computer and internet usage than those in towns and villages, which determines the feasibility of e-learning (38). Previous research found that inadequate access to technology, studying materials, and computers can leave students marginalized and anxious, affecting online learning (39). At the same time, students are more satisfied with the teaching method of combination of live and record, which reduces the problem of poor network connection to a certain extent and helps students to after-school review (40). It is recommended that improving the Information and Communication Technology infrastructure is warranted in the long run. Meanwhile, using and developing the offline curriculum resources is essential in enhancing E-learning adaptability. Colleges and universities should assess the difficulties in E-learning from rural nursing students so that alternate study plans and strategies can be worked out in advance.

It was of great interest to discover that duration of classes can be used as one of the significant predictors in E-learning adaptability. Similar findings to those in a previous study found that most of the students prefer the duration of each class to be capped at a maximum of 45 min, which could be attributed to the maximum attention span, a student can have (41). Previous studies have shown that health issues are more common in those students who have to attend classes for >4 h a day and have long courses. It may result from prolonged exposure to digital gadgets, which is associated with various ailments, such as digital eye strain and cervical spondylosis (42). Therefore, it is crucial that colleges and universities set aside guidelines for online teachings, such as the number of classes per day, length, and break between classes. Time-to-effort optimization and inclusion of practices, such as adopting creative and blended learning will make E-learning more effective.

## Limitations

This study used only quantitative measures rather than a mixed-methods approach. In addition, this study used only self-reported measure tools rather than e-learning proficiency within the concept of successful adaptation, such as learning outcomes. Further exploration adding objective evaluation indicators and using qualitative methods could have acquainted more comprehensive situations to learning about E-learning adaptability among nursing students.

## Conclusion

Our study identified that the E-learning adaptability of nursing students during the COVID-19 was at a moderate level. Professional identity played a significant role in the E-learning adaptability of nursing students. Educational level, residence, duration of classes, preferred teaching method, and network environment were significant predictors of the E-learning adaptability of nursing students. The conclusions of this study can provide a reference, which will improve the E-learning adaptability of nursing students and reduce the negative impact.

## Data Availability Statement

The original contributions presented in the study are included in the article/supplementary material, further inquiries can be directed to the corresponding author.

## Ethics Statement

The studies involving human participants were reviewed and approved by Research Ethics Committee in Kunming Medical University. The patients/participants provided their written informed consent to participate in this study. Written informed consent was obtained from the individual(s) for the publication of any potentially identifiable images or data included in this article.

## Author Contributions

HW and MY contributed to the conception and design of the study. HW undertook the statistical analysis and wrote the first draft of the manuscript. Both authors contributed to manuscript revision, read, and approved the submitted version.

## Funding

This study was financially supported by the Teaching Research and Reform Program of Kunming Medical University (2020-JY-Y-094).

## Conflict of Interest

The authors declare that the research was conducted in the absence of any commercial or financial relationships that could be construed as a potential conflict of interest.

## Publisher's Note

All claims expressed in this article are solely those of the authors and do not necessarily represent those of their affiliated organizations, or those of the publisher, the editors and the reviewers. Any product that may be evaluated in this article, or claim that may be made by its manufacturer, is not guaranteed or endorsed by the publisher.

## Figures and Tables

**Appendix Table 1:** Multiple comparisons of the one-way ANOVA for E-learning adaptability of nursing students (*N* = 585).

**Variable**	**(I)group**	**(J)group**	**Mean difference (I–J)**	**Std. Error**	***P***
Duration of classes in a week	<15 h	15**–**25 h	–0.336	0.039	0.000***
		>25 h	0.168	0.055	0.007**
	15**–**25 h	<15 h	0.336	0.039	0.000***
		>25 h	0.504	0.056	0.000***
	>25 h	<15 h	–0.168	0.055	0.007**
		15**–**25 h	–0.504	0.056	0.000***
Preferred teaching method	Record	Live	–0.062	0.055	0.499
		Combination	–0.417	0.108	0.001**
	Live	Record	0.062	0.055	0.499
		Combination	–0.355	0.098	0.002**
	Combination	Record	0.417	0.108	0.001**
		Live	0.355	0.098	0.002**

**Significant at 0.01, ***Significant at 0.001. ANOVA, Analysis of Variance.

**Appendix Table 2:** Dimensions and total scores of E-learning adaptability and professional identity (*N* = 585).

**Variable**	**Dimensions**	**Items**	**Mean (SD)**
Undergraduates' learning adaptability questionnaire (ULAQ)	2	Learning motivation	3.34 (0.56)
		Learning behavior	3.42 (0.52)
		Total scores	3.41 (0.51)
Professional Identity Questionnaire for Nurse Students (PIQNS)	5	Professional self-image	22.55 (4.03)
		Benefits of retention and risk of turnover	13.54 (2.88)
		Social comparison and self-reflection	11.28 (2.15)
		Independence of career choice	6.39 (1.51)
		Social modeling	7.85 (1.60)
		Total scores	61.60 (10.06)
